# Proteomic characterization of head and neck paraganglioma and its molecular classification

**DOI:** 10.3389/fnmol.2024.1391568

**Published:** 2024-08-21

**Authors:** Xi Wang, Jiameng Sun, Guodong Feng, Xu Tian, Yang Zhao, Zhiqiang Gao, Wei Sun

**Affiliations:** ^1^Department of Otorhinolaryngology, Peking Union Medical College Hospital, Chinese Academy of Medical Sciences and Peking Union Medical College, Beijing, China; ^2^Beijing National Laboratory for Molecular Sciences, Key Laboratory of Analytical Chemistry for Living Biosystems, Institute of Chemistry, Chinese Academy of Sciences, Beijing, China; ^3^University of Chinese Academy of Sciences, Beijing, China; ^4^Institute of Basic Medical Sciences, School of Basic Medicine, Peking Union Medical College, Chinese Academy of Medical Sciences, Beijing, China

**Keywords:** head and neck paraganglioma, proteome, function annotation, molecular classification, pathogenic mechanism

## Abstract

**Background:**

Head and neck paragangliomas (HNPGLs) are rare neuroendocrine tumors that pose significant challenges in both diagnosis and treatment. The pathogenic mechanism remains unclear, and there is no proteomic analysis-based molecular classification. Therefore, gaining a deeper understanding of this disease from the protein level is crucial because proteins play a fundamental role in the occurrence and development of tumors.

**Methods:**

We collected 44 tumor samples from patients diagnosed with HNPGL. The adrenal paraganglioma tissue (*N* = 46) was used as the disease control group and the chorda tympani nerves (*N* = 18) were used as the control group. High-pH reversed-phase liquid chromatography and liquid chromatography with tandem mass spectrometry analyses were used to build an integrated protein database of tumor samples. We then obtained two sets of differentially expressed proteins between the tumor group and the control group to identify the unique proteomic signatures of HNPGLs. Ingenuity pathway analysis annotations were used to perform the functional analysis. Subsequently, we developed a clinically relevant molecular classification for HNPGLs that connected the clinical characteristics with meaningful proteins and pathways to explain the varied clinical manifestations.

**Results:**

We identified 6,640 proteins in the HNPGL group, and 314 differentially expressed proteins unique to HNPGL were discovered via inter-group comparison. We identified two HNPGL subgroups that significantly differed in clinical manifestation and proteomic characteristics. On the basis of the proteomic results, we proposed a pathogenic mechanism underlying HNPGL.

**Conclusion:**

We conducted a comprehensive analysis of the molecular mechanisms of HNPGL to build, for the first time, a clinically relevant molecular classification. By focusing on differential proteomic analyses between different types of paragangliomas, we were able to obtain a comprehensive description of the proteomic characteristics of HNPGL, which will be valuable for the search for significant biomarkers as a new treatment method for HNPGL.

## 1 Introduction

Pheochromocytomas (PCCs) and paragangliomas (PGLs), collectively referred to as PPGLs, are rare neuroendocrine tumors (Opocher and Schiavi, 2010). Head and neck PGLs (HNPGLs) account for 65–70% of all PGLs and approximately 0.6% of all head and neck tumors (Amar et al., 2005). Research conducted by the World Health Organization in 2022 revealed that the incidence of HNPGL is approximately 0.3–1/100000 (Amar et al., 2005; Opocher and Schiavi, 2010; Wohllk et al., 2010). Depending on the development site, HNPGLs can be divided into several major categories: carotid body tumor, glomus jugular tumor, glomus tympanicum tumor, and vagal PGL. Carotid body tumors are the most common type of HNPGL and occur in the carotid body at the bifurcation of the common carotid artery. These tumors are usually benign (Wohllk et al., 2010; Fishbein, 2016; Lotti et al., 2019). The clinical manifestation of HNPGLs is diverse, and the rate of early missed diagnosis is high. Consequently, most patients fail to receive timely and optimal treatment, which affects prognosis. Furthermore, patients who undergo surgical treatment encounter various problems, such as high surgical risk and a negative effect on quality of life post-surgery (Jafri and Maher, 2012). Moreover, the cost of clinical treatment is extremely high. Owing to the size of the Chinese population, the number of PGL patients in China is considerable and thus cannot be ignored. However, the specific pathogenesis of HNPGL at the protein level remains unclear.

Previous studies have shown that the germline mutations of genes encoding succinate dehydrogenase (SDH) subunits or cofactors (e.g., SDHA, SDHB, SDHC, SDHD, and SDHx) are the major contributors to familial HNPGLs (Wohllk et al., 2010; Fishbein, 2016). Additionally, 30% of all HNPGL patients could be tested for germline mutations in SDHx (Jafri and Maher, 2012; Lotti et al., 2019), and patients who do not have a clear family history, which is known as the “sporadic type,” could also carry this mutated gene (Chen et al., 2017). SDHD mutations possess an autosomal dominant transmission feature with a parent-of-origin effect (Calsina et al., 2019). Studies have revealed that paternally imprinted tumor suppressor genes, mapped to the 11p15 region, synergize with SDHD mutations to promote tumorigenesis in PGLs (Tsoli et al., 2021). Maternal transmission of the SDHD mutation is rare in patients with PPGLs; as of 2022, there had only been three cases reported (Baysal et al., 2000; Zhu et al., 2015; Evenepoel et al., 2018). Despite the extremely low incidence, these findings are of great significance for understanding the pathogenesis of familial HNPGL. Recently, it was reported that promoter methylation of SDHC may also contribute to the inactivation of SDH, which, in turn, results in the development of PPGLs (Crona et al., 2017; Janssen et al., 2017). Thus, DNA methylation-based epigenetic markers have become target biomarkers for certain tumors; moreover, epigenetic therapies have also shown promise as a novel treatment for solid tumors (Khatami et al., 2018). Chiara and colleagues found that the epigenetic modification of SDHC was supplemented with the heterozygous defeat of the chromosomal region, which was verified by the Knudson two-hit tumorigenesis model (Baysal, 2008). Wu et al. reported that the progressive behaviors of SDH-mutated HNPGLs, especially the SDHB-mutated type, are likely related to the inactivation of multiple tumor suppressor genes (Favier et al., 2015). Rao et al. demonstrated that the accumulation of oncometabolites inhibits 2-oxoglutarate-dependent dioxygenases, which activate the pseudohypoxic pathway and lead to the occurrence of PGL. Multiple biological processes are influenced by this pathway, which includes tumorigenesis, angiogenesis, apoptosis, cell migration, and metastatic spread (Maria et al., 2015). However, to date, there have not been any proteomic studies of HNPGL. Furthermore, there are limited studies focused on the proteomic characteristics of HNPGL and functional disease analyses. Proteomics-related technology has made significant progress in recent years, enabling a deeper understanding of the pathogenic mechanisms underlying diseases at the protein level. Previous studies have also suggested that the potential pathogenesis of PGL is related to immune response, respiratory electron transfer disorder, and inflammatory disorder. Therefore, we aimed to focus on the function and enriched pathways of disease differential proteins in various tumor and control groups.

We collected 44 tumor samples from patients diagnosed with HNPGL and performed functional analyses to identify the proteomic features of HNPGL (Group H, *N* = 44). We then used an adrenal PGL group (Group A, *N* = 46) as the disease control group and the chorda tympani nerve group (Group C, *N* = 18) as the control group. A comparison between the tumor and control groups revealed two groups of differentially expressed proteins (DEPs). We then conducted ingenuity pathway analysis (IPA) of these two sets of DEPs to determine the functional and canonical pathway differences between the two types of PGLs. Our main objective was to gain a better understanding of the different pathologies of HNPGLs by evaluating the functions and enriched pathways of the DEPs.

We aimed to build a comprehensive profile of the proteomic features of HNPGL and provide thorough functional annotations. We applied differential proteomic analysis to obtain a comprehensive perception of HNPGL and its significant biomarkers. Our findings will deepen our understanding of the HNPGL proteome and provide valuable data to support clinical practice.

## 2 Materials and methods

### 2.1 Ethical approval

Before study enrollment, all participants and their family members were verbally informed about the study. All participants provided written informed consent. The consent forms and research protocol were officially reviewed and approved by the Ethics Committee for Human and Animal Research at Peking Union Medical College (no. I-22PJ057).

### 2.2 Clinical materials

#### 2.2.1 Obtaining fresh tissue

Tumor samples were collected from patients who had undergone surgery at our otorhinolaryngology department. The final diagnosis was pathologically confirmed following the surgery. In total, 108 samples were obtained, of which 44 were from HNPGL patients, 46 were from patients with adrenal PGL, and 18 were chorda tympani nerves from non-tumor patients who received otology surgery and whose chorda tympani nerve could not be preserved or had to be removed. After the tumor was surgically removed, the samples were cut into small 1-cm^3^ blocks and washed in saline three times to remove blood contamination. The tissue was immediately placed in a tissue processing cassette and sent to the laboratory for further processing.

#### 2.2.2 Fixation

We added 10% neutral formalin to the tissue samples at 20 times the tissue volume. An adequate volume of fixative (with a ratio of 20:1) was used to avoid twisting of the fresh specimen and ensure reliable fixation. The total fixation time for the specimen was between 6 and 48 h.

#### 2.2.3 Dehydration

The water was removed from the specimen before wax infiltration. The samples were placed in 80% ethanol and 90% ethanol for 1 h successively, followed by 95% ethanol for 2 h and 100% ethanol for 3 h.

#### 2.2.4 Clearing

We used xylene as the clearing agent. Samples were placed in xylene for 2 h.

#### 2.2.5 Wax infiltration

To enable the cutting of samples, we applied high-quality wax for infiltration. The total time required for wax infiltration was 3 h.

#### 2.2.6 Embedding

Tumor samples were carefully orientated in a mold filled with molten wax. A clean cassette was then placed on top of the mold, and additional wax was added. This was then placed on a cold plate for solidification. The block and the attached cassette were then removed from the mold and prepared for microtomy.

### 2.3 Protein sample preparation

All samples were deparaffinization before protein extraction. The FFPE tissues were deparaffinized by three incubations with 1 mL xylene. After brief vortexing, the samples were centrifuged at 12,700 × g for 10 min, and the supernatant was discarded. The samples were then rehydrated by incubation with 1 mL of 100, 80, and 70% ethanol sequentially, followed by air drying for 10 min under a fume hood (Friedrich et al., 2021).

The dewaxed FFPE samples were then carefully scraped off and placed in the tubes with 100 μL lysis buffer (5 mM ZWITTERGENT ® 3–16 Detergent, 1 mM EDTA, 10 mM Tris). The lysate was boiled at the temperature is 100°C for additional 40 min. Protein concentration was digested using a filter-aided sample preparation method (FASP) as follows: Proteins were reduced by adding DTT and heating at 95°C for 10 min. After cooling, IAM was added for alkylation and incubated in the dark for 45 min at room temperature. Then, proteins were transferred to a 30 kDa membrane for further digestion. Trypsin was added to the proteins at a ratio of 1:50, and the mixture was incubated at 37°C for 8 h (Wiśniewski et al., 2009). The tryptic peptides were collected for further LC–MS analysis.

### 2.4 LC–MS/MS analysis

The digested peptides were analyzed on the Ultimate3000 LC system (Thermo Fisher Scientific, USA) using an integrated capillary LC column (75 μm × 50 cm), and maintained at a constant column temperature of 60°C throughout the entire experiment. Peptides were separated in 25 min at a flow rate of 1.5 μL/min with a step linear solvent gradient. Mobile phases A and B were water with 0.1 vol% formic acid and acetonitrile with 0.1% v/v formic acid, respectively. The gradient was set as follow: mobile phases B was first increased from 5 %to 20% in 15.5 min, then increased from 20 to 30% in 5 min, and increased from 30 to 50% in 1 min, then increased to 90% in 0.1 min, and maintained for 1.3 min, and finally ramped from 90 to 5% in 0.1 min, and maintained for 2 min.

Liquid chromatography was coupled online to a hybrid TIMS quadrupole TOF mass spectrometer (Bruker timsTOF Pro2) via Captive Spray nano-electrospray ion source. The dual TIMS analyzer was operated at a fixed duty cycle close to 100% using equal accumulation and ramp times of 80 ms each. Linear calibration of TIMS elution voltages using three Agilent ESI-L Tuning Mixed ions (m/z 622, 922 and 1,222) to obtain ion mobility reduction coefficient (1/K0). All samples were analyzed with parallel accumulation–serial fragmentation combined with data-independent acquisition (dia-PASEF) in positive mode. To perform DIA, we extended the instrument control software (Bruker otofControl) to define quadrupole isolation windows as a function of the TIMS scan time (dia-PASEF). Therefore, 32 mass steps per cycle were build up. Singly charged precursors were excluded by their position in the m/z–ion mobility plane, and precursors were dynamically excluded for 0.25 min. A scan range was set from 350 to 1,200 m/z. The collision energy was ramped linearly as a function of the mobility from 59 eV at 1/K0 = 1.6 Vs cm^−2^ to 20 eV at 1/K0 = 0.6 Vs cm^−2^.

### 2.5 Data analysis

All raw data were imported into Spectronaut 16 and searched against the human Swissprot database (*Homo sapiens*, 20,358 Swissprot, 2019_05 version) appended with the indexed retentions time fusion protein sequence (Biognosys). The DIA-MS data were analyzed using Spectronaut Pulsar (Biognosys, Switzerland) with default settings, which was applied in this study’s previous research (Proteomic Study of Aqueous Humor and Its Application in the Treatment of Neovascular Glaucoma) (Yu et al., 2020). Spectronaut identified and quantified proteins by matching retention time, m/z, and other parameters to a peptide library. The retention time prediction type was configured as dynamic indexed retention time, and interference correction at the MS2 level was activated. Both MS1 and MS2 tolerance strategies were set to dynamic, and a correction factor was applied to the automatically determined mass tolerance, with the correction factor for MS1 and MS2 both set to 1. The precursor posterior error probability cutoff was established at 1, with precursors not meeting this cutoff being imputed. For quantification calculations, the top *N* (min: 1; max: 3) precursors per peptide were utilized, with their ranking order determined by a cross-run quality measure. Peptide intensity was calculated by summing the peak areas of their respective fragment ions for MS2. Cross-run normalization was employed to correct for systematic variance in LC–MS performance using a local normalization strategy. This normalization assumed that, on average, a similar number of peptides are upregulated and downregulated, with the majority of peptides in the sample remaining unregulated across runs and retention times. All results were filtered using a Q value cutoff of 0.01, corresponding to an FDR of 1%.

### 2.6 Bioinformatics analysis

#### 2.6.1 Differential expression analysis of the proteome

Differential proteomic analysis was performed for 6,640 quantified identified proteins from a total of 7,139 proteins detected. We conducted Wilcoxon tests to identify dysregulated proteins that significantly differed between HNPGL (Group H) and adrenal PGL patients (Group A). *p*-values were corrected using the Benjamini and Hochberg procedure. Significantly up- or down-regulated proteins were defined using an adjusted *p*-value <0.01 and | log2 (fold change) | >1.5. In addition, we used limma analysis to adjust for clinicopathological characteristics. We also compared the DEPs between two tumor groups with the healthy tympanic nerve group (Group C) separately using the same methods.

#### 2.6.2 Functional enrichment analysis

We used the Panther database^1^ to allocate gene symbols to the different sets of DEPs. The Panther database is a comprehensive resource that offers gene symbol annotations based on protein sequences. This classification provides meaningful information on the roles and functions of the DEPs in numerous biological processes. To further analyze protein–protein interactions, we uploaded all identified DEP sets to the STRING database^2^ and examined protein connections and functional relationships. A minimum interaction score of 0.5 was used to ensure the reliability of the expected interactions. We also apply OPLS-DA (Orthogonal Partial Least Squares Discriminant Analysis) for having a better understand relationships between variables in classification and distinguishing differences between different categories. OPLS-DA is a statistical analysis method derived from Partial Least Squares (PLS) regression. It begins by applying Partial Least Squares regression to perform dimensionality reduction and predictive modeling on the data. OPLS introduces an orthogonal component that separates the data into predictive and orthogonal parts, thereby enhancing model interpretability and predictive performance.

To further analyze the DEPs, we used Ingenuity Pathway Analysis (IPA) software. IPA is a powerful tool for exploring biological pathways, diseases, and functional annotations, and is widely used in bioinformatic analysis. The accession protein numbers were uploaded to IPA for further analysis. The results were ranked according to *p*-value or -log *p*-value. The order represents the importance of the relationship between the DEPs and the pathways or functions.

### 2.7 Statistical analysis

Similar to the functional analysis, DEPs were defined as up-regulated when abundance was increased ≥1.5-fold or down-regulated when abundance was decreased ≤0.67-fold relative to each group. These are the same thresholds used in Section 2.6.1. We reviewed the abundance alteration of peptides and set a cutoff value of 1.5-fold change relative to the tumor group. An adjusted *p*-value of <0.05, using the Benjamini and Hochberg method, was applied to identify meaningful differential peptides.

## 3 Results

### 3.1 Quality control

**Figure 1:**
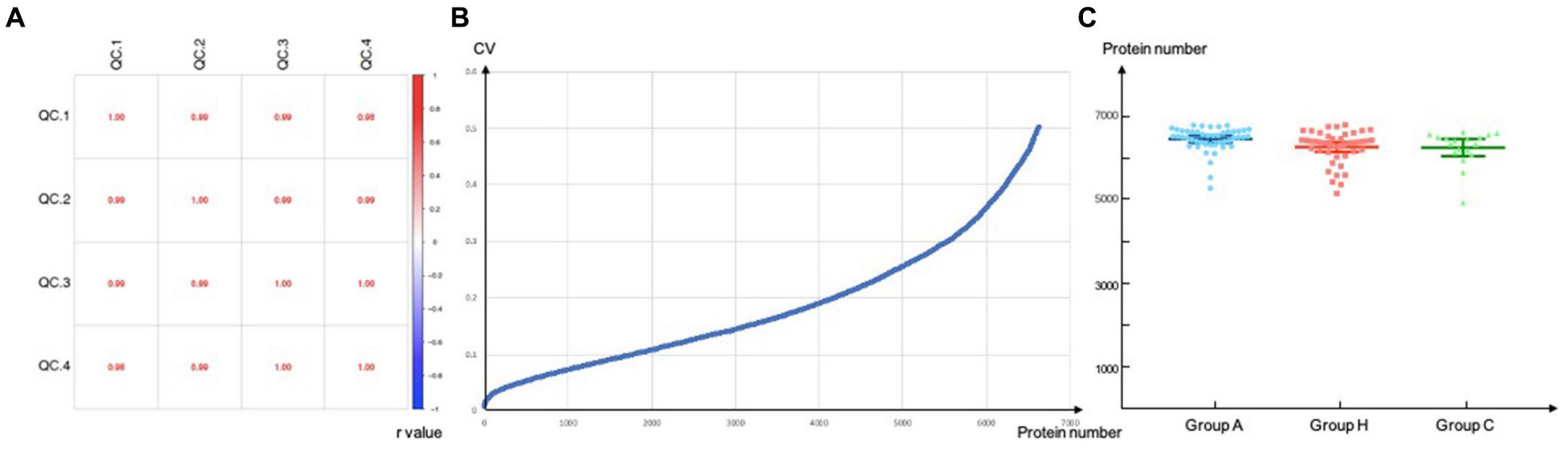
Quality control. Correlation of QC samples. QC, quality control. **(A)** Reproducibility of proteins of DIA-MS analysis. Values inside the boxes represent Pearson correlation coefficient (r value). r value tends to 1/-1 suggested high correlation while r value tends to 0 refers to low correlations; **(B)** CV, coefficient of variation, a total of 6640 proteins were screened when the CV threshold set to 0.5; **(C)** Number of proteins identified per group.

We initially included 47 patients with adrenal PGL, 44 patients with HNPGL, and 18 healthy chorda tympani nerve samples. We collected healthy chorda tympani nerves from non-tumor patients who had undergone surgery in which the chorda tympani nerve was surgically resected or could not be preserved during surgery. To ensure the stability and reliability of the experiment results, we first carried out quality control on the collected samples. Because the tumor and nerve tissues were directly obtained via surgery, we evaluated the plasma contamination of the samples. We calculated the proportion of plasma peak protein in 30 samples and estimated that the average proportion of plasma peak protein in 108 samples was 8.66% with a standard deviation of 3.64. On the basis of these calculations, we excluded one sample with serious plasma contamination (defined as exceeding 3 standard deviations), which had a proportion of peak plasma protein of 31.79%. We then calculated the batch effect and the number of detected proteins to verify the stability of the MS system and found that there was no significant difference in the number of proteins detected among the three groups of sample systems (Figure 1). This resulted in the inclusion of a total of 108 samples, of which 46 were from adrenal PGL patients (Group A), 44 were from HNPGL patients (Group H), and 18 were healthy chorda tympani nerve samples (control Group C).

Correlation of QC samples. QC, quality control. (a) Reproducibility of proteins of DIA-MS analysis. Values inside the boxes represent Pearson correlation coefficient (*r* value). *r* value tends to 1/−1 suggested high correlation while *r* value tends to 0 refers to low correlations; (b) CV, coefficient of variation, a total of 6,640 proteins were screened when the CV threshold set to 0.5; (c) Number of proteins identified per group.

### 3.2 Clinical results

Among the 46 patients diagnosed with adrenal PGL, 18 were men and 28 were women, with an average age of 48.26 years (± 13.43 years). Forty-four patients (95.65%) had endocrine dysfunction; the most common clinical manifestation was blood pressure fluctuations. Nine patients had multiple lesions and were diagnosed as multiple endocrine neoplasia type 2. Among the 44 patients in Group H, there were 9 men and 35 women, with an average age of 46.16 years (± 12.62 years). Of these, 6 patients had lymph node metastasis, 9 patients had endocrine dysfunction via catecholamine secretion, 33 patients had a single set tumor, and the remaining 11 patients had multiple lesions (Table 1).

**Table 1:** Detailed clinical information of 46 patients with adrenal PGL (group A) and 44 patients with HNPGL (group H).

		Group A^a^	Group H^b^
Sexr^c^		1:1.57	1:3.89
Age		48.26 ± 13.43	46.16 ± 12.62
Lymph invasion	Yes	6	6
	No	40	38
Endocrine function	Yes	44	9
	No	2	35
Original site	Single	37	33
	Multiple	9	22
MEN 2^d^	Yes	9	/
	No	37	/

a Adrenal PGL group.

b HNPGL group.

c Male to female ratio.

d Multiple endocrine neoplasia type 2.

### 3.3 PGL proteome analysis workflow

**Figure 2:**
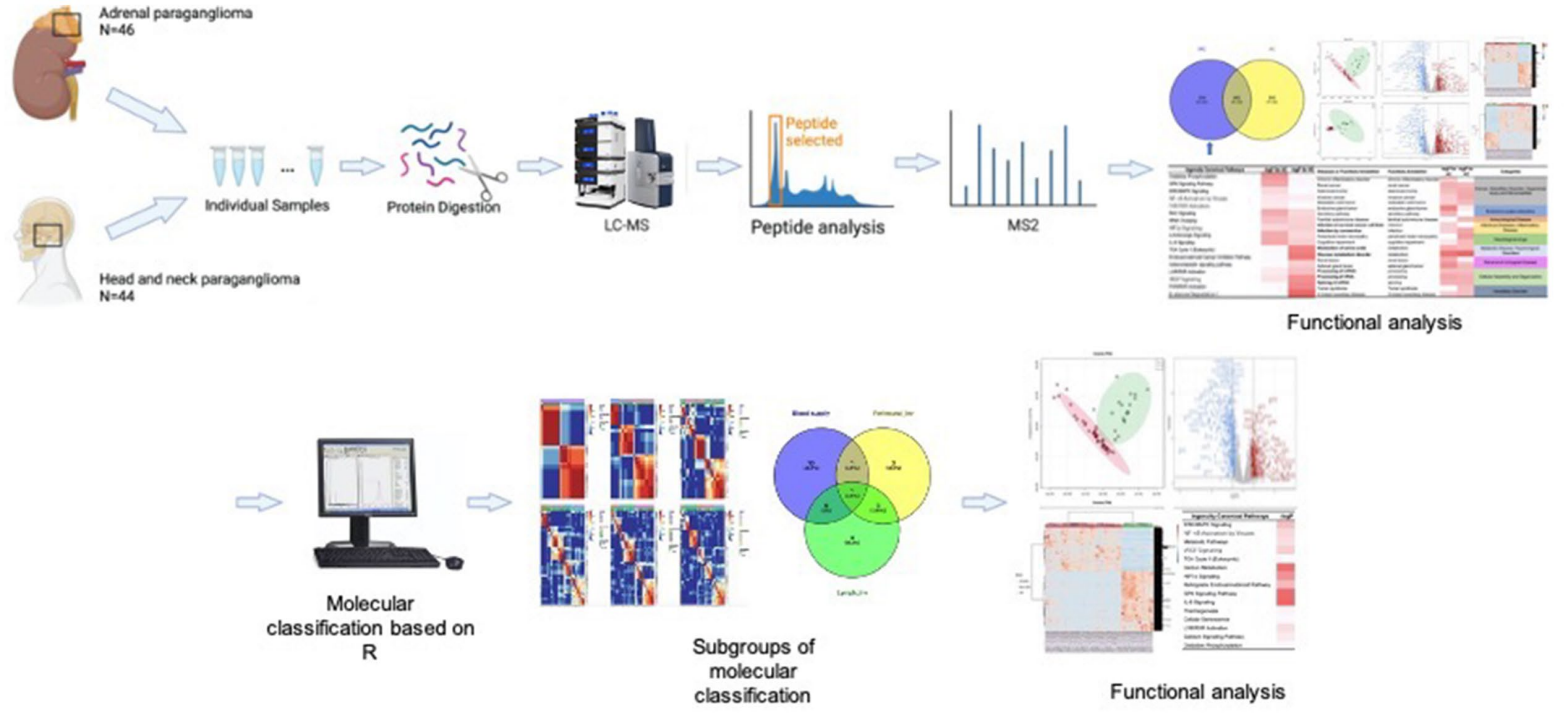
Workflow.

All PGL samples (i.e., Groups A and H) were prepared as individual samples and subsequently digested and evaluated using LC–MS/MS. Because PGLs are tumors that originate from nerves, we collected healthy chorda tympani nerves as a healthy control for IPA (Figure 2).

**Figure 3:**
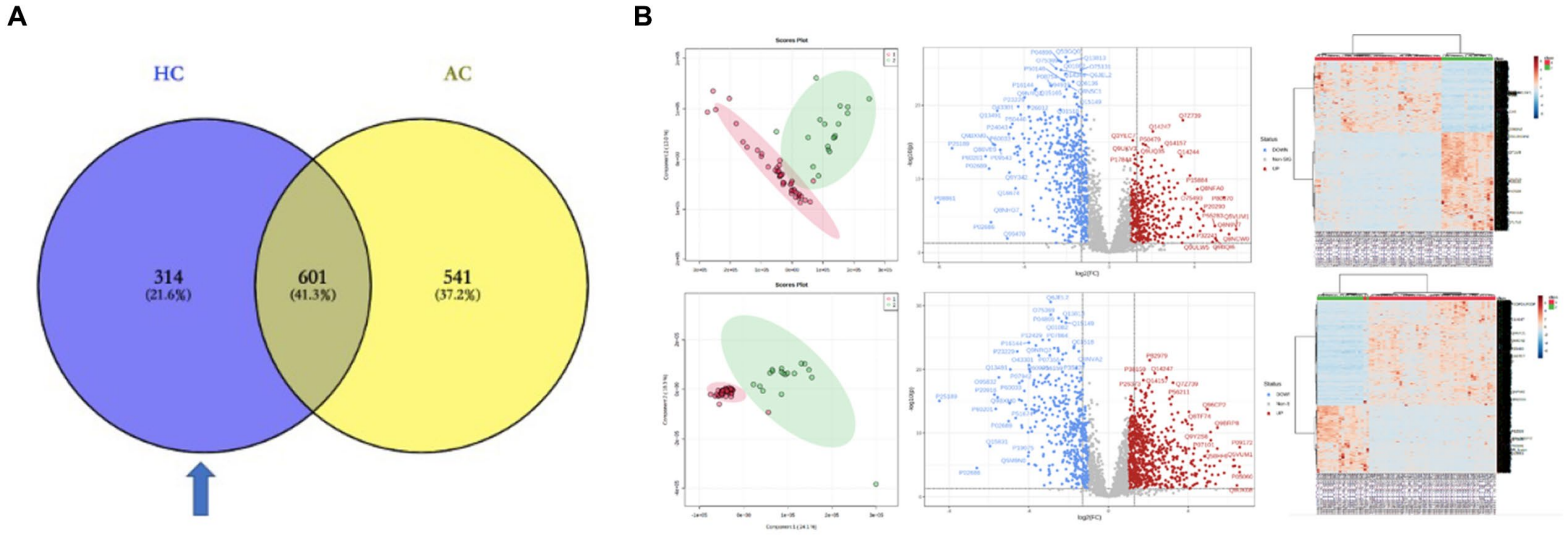
DEPs of the two tumor groups and the healthy nerve control. **(A)** DEPs between the tumor groups and control group. Blue region refers to the set of proteins specific to HNPGL. **(B)** Principal component analysis (PCA), volcano plots, and heatmaps for the DEPs between the two tumor groups and control group. HC, DEPs between Group H and Control; AC, DEPs between Group A and Control.

To explore the pathogenesis of PGL and the specificity of HNPGL, we compared each tumor group with healthy chorda tympani nerves to obtain two sets of DEPs. We then used the intersection of these two sets of DEPs to identify a specific set of proteins for HNPGL, as shown in Figure 3A. Five proteins underwent immunohistochemistry to validate the quantification of the target proteins.

### 3.4 Proteomics analysis of tumor groups compared with healthy controls

The LC–MS/MS analysis of the tumor and nerve samples identified a total of 7,139 proteins, of which 6,640 proteins were quantitatively identified (unique peptides ≥2). The proteomics analysis with tandem mass identified 1,142 proteins that were significantly differentially expressed in the adrenal PGL group compared with the control group. Among these proteins, 699 and 443 proteins were up- and down-regulated, respectively (Figure 3B). Using the same methods, we identified 915 DEPs between the HNPGL and control groups, of which 385 were up-regulated and 530 were down-regulated (Figure 3B). In the HNPGLs, nicotinamide adenine dinucleotide dehydrogenase 1 alpha subcomplex 2, RAB5C, nicotinamide nucleotide transhydrogenase, and nicotinamide adenine dinucleotide dehydrogenase 1 alpha subcomplex 10 were significantly up-regulated, whereas superoxide dismutase 3, periaxin, and peripheral myelin protein 2 were down-regulated.

**Figure 4:**
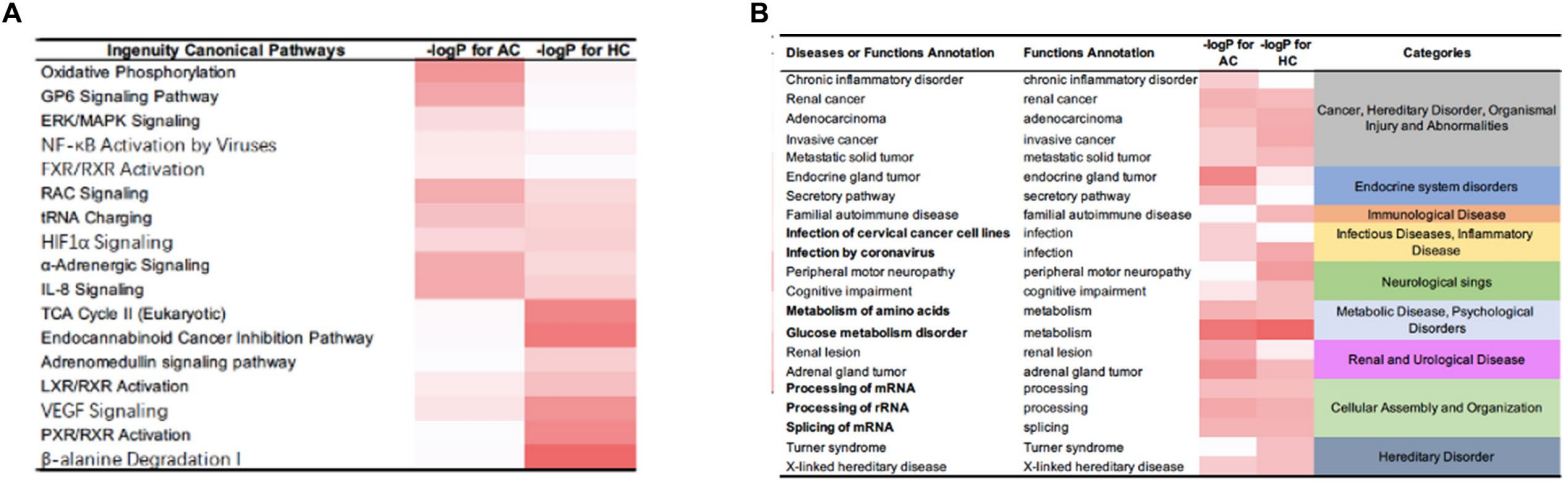
IPA results for the DEPs between tumor groups and control. **(A)** Ingenuity canonical pathways. **(B)** Disease and functions annotation.

IPA identified certain pathways that were associated with PGL. Although some of the pathways were related to both tumor types, there were also some pathway differences between the two groups (Figure 4). DEPs of adrenal PGL were significantly enriched in ingenuity canonical pathways, such as the oxidative phosphorylation, glycoprotein VI signaling, interleukin-8 signaling, RAC signaling, and transfer RNA charging pathways. The DEPs of HNPGLs were enriched in the tricarboxylic acid (TCA) cycle II (eukaryotic), endocannabinoid cancer inhibition, and pregnane X receptor/retinoid X receptor activation pathways, whereas the DEPs of adrenal PGL were not significantly enriched in any of these pathways. Additionally, both types of PGLs were enriched in the pseudohypoxic (hypoxia-inducible factor [HIF]-1α signaling) and nuclear factor kappa B activation signaling pathways, which suggests that these pathways are associated with the pathogenesis of PGLs, as shown in Figure 4A.

Furthermore, the IPA revealed diseases and function annotations, as shown in Figure 4B. We found that the DEPs of both adrenal PGL and HNPGL were related to the categories of cancer and hereditary disorders (i.e., chronic inflammatory disorder, invasive cancer, and metastatic solid tumor), metabolic diseases (i.e., metabolism of amino acids and glucose metabolism disorder), and cellular assembly and organization (i.e., processing of messenger RNA [mRNA] and ribosomal RNA and splicing of mRNA). Both types of PGL were also related to X-linked hereditary diseases, which belong to the category of hereditary disorders. Proteins of adrenal paraganglioma were significantly enriched in endocrine system disorders, which include the function annotations of endocrine gland tumors and secretory pathways. In contrast, proteins of HNPGL were related to neurological signs, such as peripheral motor neuropathy and cognitive impairment, which were not found in adrenal paraganglioma proteins. In addition, proteins involved in immunological diseases were found in the HNPGL group only, which suggested that the pathogenesis of HNPGL is related to immune system disorders. Proteins involved in metabolic diseases and cellular assembly and organization were found similarly across both groups, which indicated similarities in the pathogenesis of the two types of PGLs.

### 3.5 Molecular classification of HNPGL

**Figure 5:**
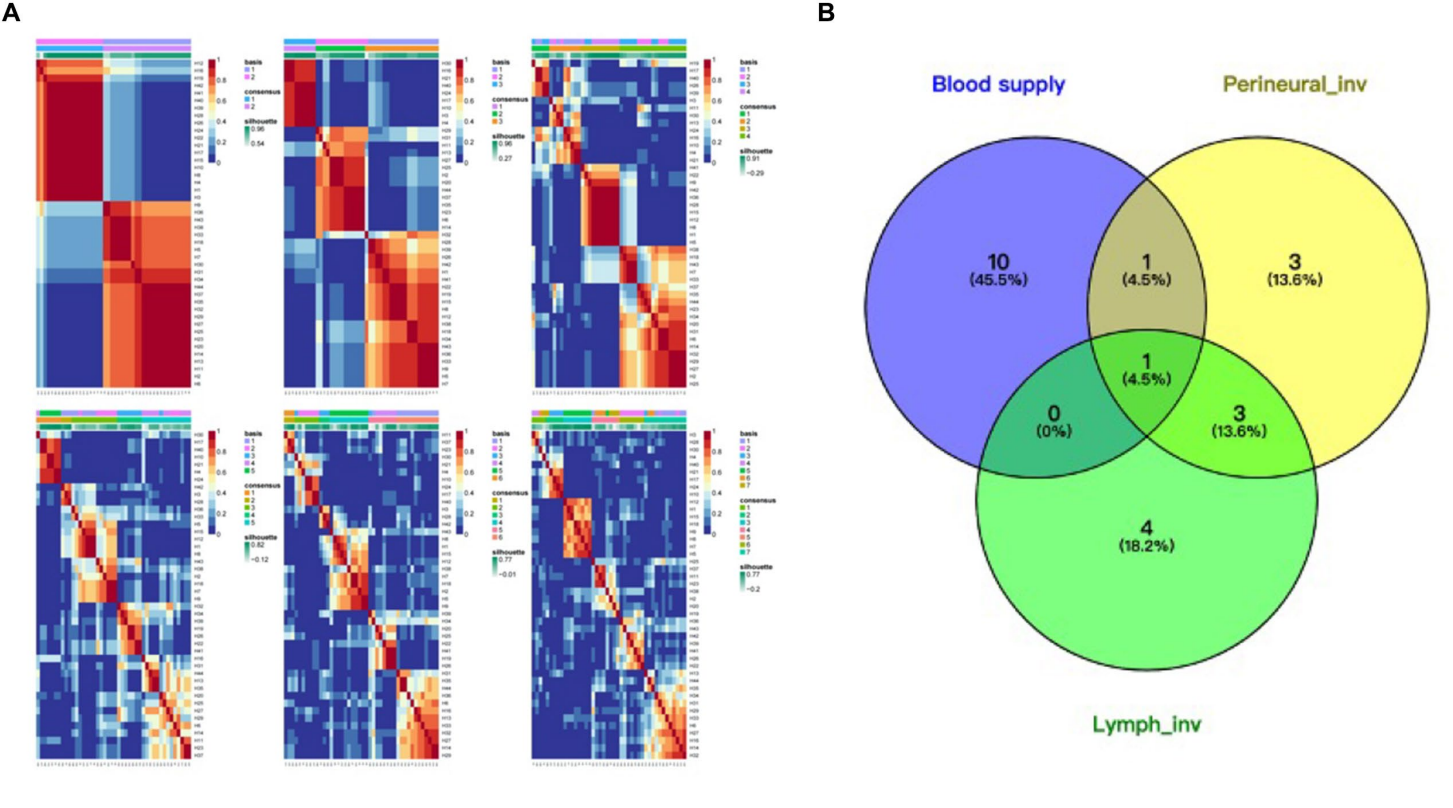
Molecular classification of HNPGL. **(A)** Different molecular classification modes, we took the first one which divided the tumor group into two subgroups. **(B)** Patients presented with different clinical characters, the blue part refers to rich in blood supply, yellow part refers to patients with perineural invasion, and green part refers to lymph invasion.

To better understand the specificity of HNPGL and link proteins with clinical manifestations, we performed molecular classification in 44 HNPGL patients, as shown in Figure 5. We first compiled meaningful clinical indicators of the 44 patients (e.g., age, sex, family history, endocrine function, progression rate, tumor volume, blood supply, lymph node, single or multiple surgeries), as shown in Table 2. We then applied the R language molecular classification algorithm to divide patients into two subgroups: Subgroup A (*N* = 19) and Subgroup B (*N* = 25). A chi-square test was used to test for differences in clinical indicators between the two subgroups (*p* < 0.05 suggests significant statistical difference). There were no significant differences in sex, age, or family history between the two subgroups. However, the clinical indicators of blood supply (*p* = 0.001), peripheral nerve invasion (*p* = 0.039), and recurrence (*p* = 0.025) were significantly different between the two subgroups (Table 2).

**Table 2:** Clinical characteristics of subgroups A and B for HNPGL.

Clinical characters		Subgroup A	Subgroup B	*p* value
Gender	Male	5 (11.4%)	4 (9.1%)	0.476
	Female	14 (31.8%)	21 (47.7%)	
Age	≤ 45y	11 (25.0%)	11 (25.0%)	0.272
	> 45y	8 (18.2%)	14 (31.8%)	
Family history	Yes	2 (4.5%)	1 (2.3%)	0.097
	No	17 (38.6%)	24 (54.6%)	
Endocrine function	Yes	5 (11.4%)	6 (13.6%)	0.566
	No	14 (31.8%)	19 (43.2%)	
Onset symptoms	Yes	15 (34.1%)	22 (50.0%)	0.416
	No	4 (9.1%)	3 (6.8%)	
Progression	Fast	6 (13.6%)	14 (31.8%)	0.107
	Slow	13 (29.5%)	11 (25.0%)	
Original site	Single	13 (29.5%)	20 (45.5%)	0.380
	Multiple	6 (13.6%)	5 (11.4%)	
Blood supply	Rich	0 (0%)	12 (27.3%)	0.001
	Poor	19 (43.2%)	13 (29.5%)	
Perineural invasion	Yes	1 (2.3%)	7 (15.9%)	0.039
	No	18 (40.9%)	18 (40.9%)	
Lymph invasion	Yes	2 (4.5%)	6 (13.6%)	0.251
	No	17 (38.6%)	19 (43.2%)	
Recurrence	Yes	0 (0%)	6 (13.6%)	0.025
	No	19 (43.2%)	19 (43.2%)	

**Figure 6:**
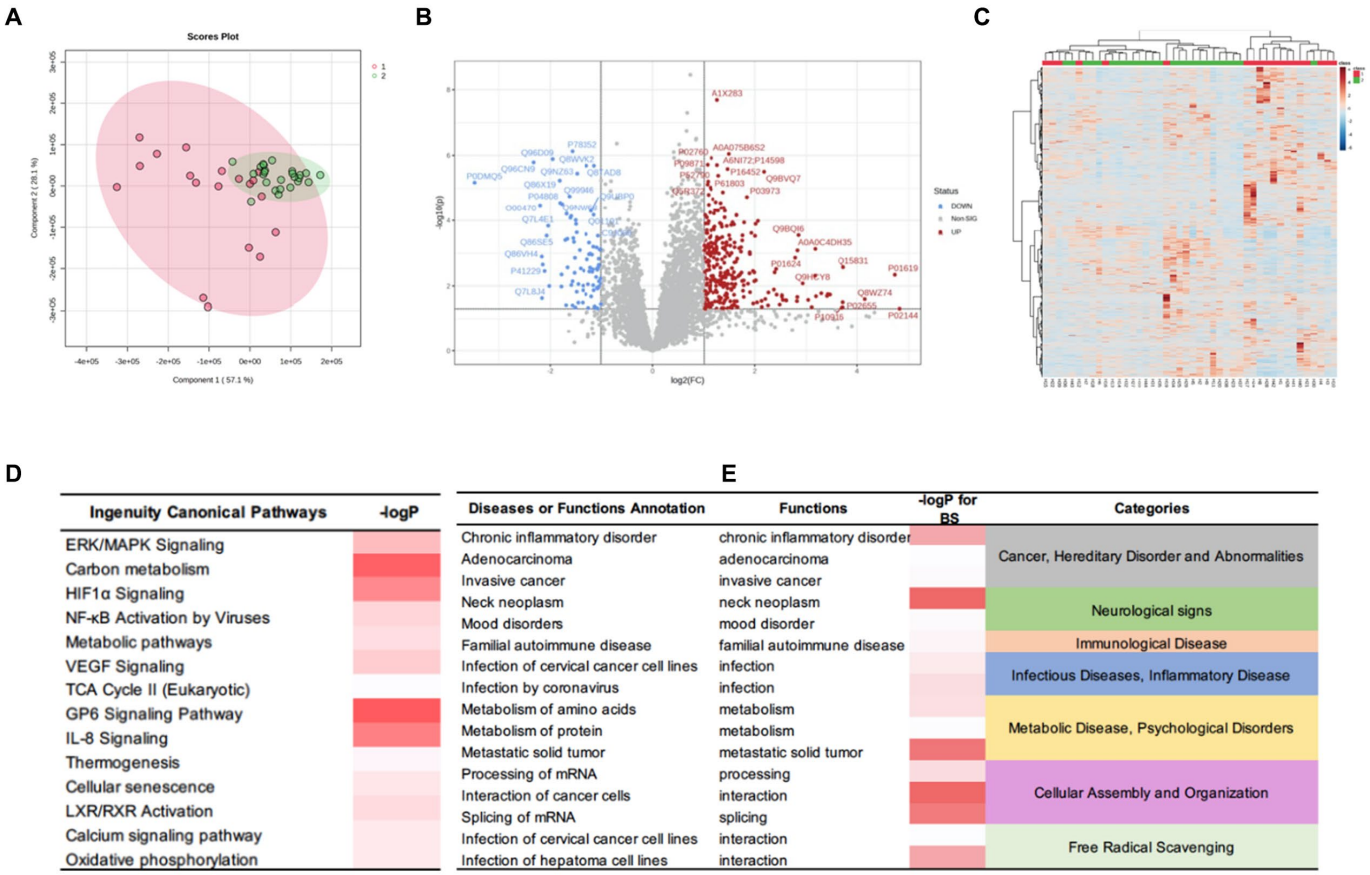
DEPs of the HNPGL subgroups and IPA results. **(A)** PCA, subgroup A are in red while subgroup B samples are in green. **(B)** Volcan plot. Sig. Down [89], the number of significantly downregulated protein is 89; Sig. Up [303], the number of significantly upregulated protein is 303; Red dots refer to upregulate protein and blue dot refers to downregulate protein. Gray dots represent for proteins which show no significant changing trend; **(C)** Heatmap for DEPs. Group 1(red) represents for subgroup A, group 2(green) represents for subgroup B. **(D)** The major canonical pathways of DEPs between two subgroups. **(E)** the diseases or functions annotation. The color of the bar in *p*-value and -logP column indicates the level of significance. IPA, ingenuity pathway analysis.

To further examine the differences between the two subgroups at the protein level, we applied the same aforementioned method to obtain a set of DEPs between the two subgroups and identified 392 DEPs. Of these, 303 were up-regulated, and 89 were down-regulated. The IPA for functional annotation identified several pathways that were distinct to each subgroup (Figure 6). The DEPs were significantly enriched in ingenuity canonical pathways, such as the extracellular signal-regulated kinase/mitogen-activated protein kinase signaling, carbon metabolism, HIF-1α signaling, TCA cycle II, cellular senescence, and metabolic pathways. The disease and function analysis showed that these proteins were related to cancer and hereditary disorders (i.e., chronic inflammatory disorder, adenocarcinoma, and invasive cancer), which is in line with the IPA results of the DEPs between the tumor and control groups. These findings suggested that these function annotations are responsible for the occurrence of PGLs. The DEPs were also found to be related to the categories of cellular assembly and organization (i.e., processing of mRNA, interaction of cancer cells, and splicing of mRNA). This may explain the differences in recurrence and peripheral nerve involvement between the two subgroups. Additionally, we revealed that the DEPs were involved in free radical scavenging, which indicated that the differences in clinical manifestation and prognosis between the two subgroups are related to abnormal changes in free radicals.

## 4 Discussion

We examined the disease characteristics of HNPGL using proteomic analysis and further explored the general characteristics and specificity of HNPGL by comparing HNPGL samples with adrenal PGL and chorda tympani nerve samples as a tumor control and healthy control, respectively. Our findings provide a comprehensive baseline for understanding the HNPGL protein map. By comparing two sets of tumor samples and healthy chorda tympani nerve samples, we were able to obtain two sets of DEPs. We revealed that these DEPs play an important role in the pathological process of HNPGL. The functional analysis of the DEPs offers valuable insight into HNPGL-related molecular mechanisms at the proteomic level and meaningful biomarkers. Identifying the proteins involved in the disease process will be vital for the development of diagnostic and treatment approaches in clinical practice.

### 4.1 Epidemiology and etiological characteristics of HNPGL

Epidemiological studies have shown that the incidence of HNPGL is low, at approximately 0.3 to 1/100000 (Amar et al., 2005; Opocher and Schiavi, 2010). Because of atypical early clinical symptoms, the rate of missed diagnosis of HNPGL is high (Jafri and Maher, 2012). Although the biological behavior of this tumor tends to be benign, the tumor often involves important nerves and blood vessels in the lateral skull base area, which results in time-consuming and difficult surgeries (Wiśniewski et al., 2009; Zhu et al., 2015; Friedrich et al., 2021). Indeed, some patients may encounter postoperative complications owing to the involvement of the posterior cranial nerves, and a series of such postoperative complications can further affect patients’ quality of life (Moosavi et al., 2019). Furthermore, some patients may be diagnosed too late and miss the window for surgical treatment (Moosavi et al., 2019; Buffet et al., 2020). Therefore, HNPGLs pose a major health threat to patients. Currently, the pathogenic mechanism underlying HNPGL is poorly understood and thus, there is a crucial need to identify molecular pathways involved in disease pathogenesis, prognostic biomarkers, and potential drug targets. This will be key to early diagnosis and treatment of HNPGL.

Several studies have investigated the molecular mechanisms underlying PPGL tumorigenesis. Mutations in SDH are responsible for nearly all familial HNPGLs (Roy and Tiirikainen, 2020), most of which are germline mutations. Several systemic reviews of somatic and germline alterations in DNA and/or RNA have shown that kinase and Wnt signaling pathways are dysregulated in PPGL (Astuti et al., 2003; Griess et al., 2020; Clifton-Bligh, 2023; Tabebi et al., 2023). Richter et al. (2016) using gene-centered copy number variation analysis in 24 patients, and Mira et al. (2018) argued that the NOTCH signaling pathway is one of the most prominently dysregulated pathways in HNPGLs. Wu and colleagues performed proteomic analysis of HNPGL samples to compare SDHB mutations and neural controls (Favier et al., 2015). Results of the gene ontology and pathway enrichment analyses showed that the oxidative phosphorylation, citric acid cycle, carbon metabolism, and metabolic pathways were dysregulated in HNPGLs. Previous reports on PPGLs also indicated that alterations in the TCA cycle, mitochondrial electron transport chain, and metabolic pathways may ultimately lead to disease (Kimura et al., 2021; Tufton et al., 2022; Bayley et al., 2023). In recent years, the hypoxia signaling pathway has also become a commonly reported mechanism involved in HNPGLs.

### 4.2 DEP functional analysis comparing PGLs and chorda tympani nerves

PCC is a neuroendocrine tumor arising exclusively from adrenal medulla chromaffin cells (Wohllk et al., 2010; Fishbein, 2016). PGLs (PGLs) are tumors of extra-adrenal chromaffin tissue located in the sympathetic or parasympathetic paraganglions. PCCs mainly synthesize, secrete, and release large amounts of catecholamines, such as norepinephrine, epinephrine, and dopamine. This causes a series of clinical syndromes, such as increased blood pressure and metabolic changes, which lead to serious diseases of the heart, brain, kidney, and blood vessels, and sometimes death (Choi et al., 2021). Correspondingly, HNPGLs typically have no endocrine function. Clinically, patients present with clinical manifestations that are caused by the compression of peripheral nerves by the tumor, or lateral skull base occupation is detected during head imaging examination (Heesterman et al., 2013). Therefore, although HNPGL and PCC both belong to the same category of PPGLs, HNPGL should be considered a distinct disease, which may be reflected in its protein components.

Because PPGLs originate from ganglia, we selected healthy chorda tympani nerves as the healthy control. We compared the HNPGL and adrenal PGL groups with the control group and obtained two sets of DEPs to determine the differences between HNPGL and adrenal PGL at the protein level. We found significant differences between groups based on the orthogonal partial least squares-discriminant analysis score plot (OPLS-DA score plot, *p* = 1.37214e-07) and heatmap analysis. We then obtained two sets of DEPs with a defined fold change of >2.0 and a *p*-value of 0.05 according to the volcano plot.

The IPA revealed 16 pathways that were considered significantly involved in the occurrence of HNPGL, and the two sets of DEPs were both related to the categories of cancer and hereditary disorders, metabolic disease, and X-linked hereditary disease. Notably, the proteins of adrenal PGL were significantly enriched in endocrine system disorders and secretory pathways, whereas the proteins of HNPGL were found to be related to peripheral motor neuropathy and cognitive impairment pathways. Additionally, the proteins involved in immunological disease were only found in the HNPGL group. These findings suggest that the two types of PGL have similar pathogenesis related to metabolic processes and cellular assembly. However, we found that the pathogenesis of HNPGL is characterized by hypoxia, immune system disorders, and nervous system damage, which highlights the possible influence of energy metabolism on the molecular processes of HNPGL.

Previous studies have reported that chronic hypoxia is a risk factor for HNPGL (Bechmann and Eisenhofer, 2021), and the pseudohypoxic pathway is related to the occurrence of HNPGL (Shuch et al., 2014). The pseudohypoxic group can be further divided into two subgroups: the first subgroup involves factors related to the TCA cycle, and the second subgroup includes Von Hippel–Lindau disease/endothelial Per-Arnt-Sim domain-containing protein 1-related genes (Movafagh et al., 2015; Bechmann and Eisenhofer, 2021). The TCA cycle consists of a group of different enzymes that connect the downstream protein to the oxidization of acetyl coenzyme A and nicotinamide adenine dinucleotide produced by the oxidization of the mitochondrial respiratory chain (Bechmann and Eisenhofer, 2021). SDH is an important protein involved in the TCA cycle (Li et al., 2018), and activation of HIF is a common feature of this cluster (Florio et al., 2018; Li et al., 2018). When cells are in a hypoxic environment, they reactively produce HIF. As a result, the HIF pathway remains continuously activated in cells, even under normal oxygenation, which leads to epigenetic alterations in HIF target genes that affect various processes, including proliferation, angiogenesis, migration, apoptosis, and invasion (Florio et al., 2018; Sato and Inomoto, 2020; Bechmann and Eisenhofer, 2021; Manotas et al., 2023). Such processes may eventually lead to the formation of PPGLs. We identified proteins related to the TCA cycle and HIF-1α signaling, which is consistent with altered protein metabolism in PGL, especially HNPGL.

In summary, our findings offer valuable insight into the differences between adrenal PGL and HNPGL at the protein level, highlighting the potential role of cellular metabolism, the TCA cycle, and HIF-1α signaling in the pathogenesis of adrenal PGL and HNPGL.

### 4.3 DEP functional analysis of the different HNPGL subgroups

To further understand the specificity of HNPGL and explore the relationship between proteins and clinical manifestations, we performed molecular classification in HNPGL patients using an R language molecular classification algorithm to divide patients into two subgroups: Subgroup A (*N* = 19) and Subgroup B (*N* = 25). We revealed significant differences between the two subgroups based on the orthogonal partial least squares-discriminant analysis score plot (*p* = 1.163741e-07) and heatmap analysis. We identified a total of 392 DEPs, of which 303 were up-regulated and 89 were down-regulated.

The functional analysis of these DEPs showed enrichment in the extracellular signal-regulated kinase/mitogen-activated protein kinase signaling, carbon metabolism, HIF-1α signaling, TCA cycle II, cellular senescence, and metabolic pathways. Our findings are consistent with functional pathways reported in previous studies. Additionally, these DEPs were related to cellular assembly and organization (i.e., processing of mRNA, interaction of cancer cells, and splicing of mRNA) and free radical scavenging. These findings suggest that the two subgroups have distinctive molecular features. Indeed, we revealed differences in tumor blood supply, peripheral nerve involvement, and recurrence between the two subgroups. Therefore, these proteins and their enriched pathways may contribute to the different clinical manifestations of the two subgroups. In particular, tumor recurrence and invasion of surrounding structures may be related to abnormal cell proliferation, migration, and adhesion.

The Wnt signaling pathway is associated with the occurrence of PGL, especially in patients with sporadic PPGL (Ercolino et al., 2008). This pathway is triggered by somatic mutations or gene fusions in cold shock domain-containing E1, affecting mastermind-like transcriptional coactivator 3 (Ercolino et al., 2008; Yeap et al., 2011), which would, in turn, activate the Wnt and Hedgehog signaling pathways (Müller, 2011). Previous studies have suggested that the Wnt pathway plays a crucial role in several biological processes, including cell proliferation, adhesion, and differentiation (Yeap et al., 2011). Therefore, such tumors would be more prone to recurrence and distant metastasis. Among the DEPs identified for Wnt pathway, there are also downstream proteins of mutated genes. This confirms the reliability of our results and also suggests that the pathogenesis of subgroup B, with poor clinical prognosis, is likely related to this pathway.

Previous studies have shown that germline mutations of genes encoding SDH subunits or cofactors are the cause of almost all familial HNPGLs (Wohllk et al., 2010; Carmona-Rodríguez et al., 2020). Genetic mutations of SDH subunits lead to abnormal electron transfer in the respiratory chain of the TCA pathway, which, in turn, leads to tumorigenesis (Pang et al., 2018; Carmona-Rodríguez et al., 2020). Several studies to verify this process have suggested that various genes and related pathways are responsible. Superoxide dismutase 3 has been shown to be related to the regulation of HIF-1α, which participates in the process of several malignancies, such as breast, lung, pancreatic, and colorectal carcinomas (Rao et al., 2013). Nicotinamide adenine dinucleotide dehydrogenase 1 alpha subcomplex 2 plays an important role in TCA, and we found that its downstream protein was significantly up-regulated in PGL samples (Fishbein et al., 2017). Previous studies have also reported that these proteins are present in patients with neurodegenerative diseases, such as Alzheimer’s disease, Parkinson’s disease, and various genetic degenerative diseases of the nervous system (Fishbein et al., 2017). We observed that this protein is up-regulated in the HNPGL group, which is consistent with the findings of Crona et al. (2017). We speculate that this protein and related pathways are responsible for the occurrence of HNPGLs via the interference of proliferation and reduction in apoptosis. In addition, the expression of complex I and IV subunits in the respiratory electron transport chain has been shown to be down-regulated in patients with HNPGL, which was verified in our data (Horton et al., 2022). Our exploration of the mechanisms underlying metabolic disorders and PPGLs provides a foundation for the development of new diagnostic and treatment strategies.

### 4.4 Application of molecular typing results in clinical practice

Currently, there is no clinical classification of HNPGLs based on differences in pathogenic genes or protein levels. Our study is the first study to molecularly classify HNPGL according to proteomics. We noted that our patients varied considerably in terms of tumor characteristics, disease progression rate, and whether they required secondary or multiple surgeries. We considered that these variations may be attributed to differences in gene mutations or protein expression. We revealed differences in several clinical characteristics between the two subgroups of HNPGL patients. The functional analysis of the DEPs enabled us to examine the reason for the differences in clinical manifestations between the two subgroups. In particular, we identified downstream DEPs related to the Wnt signaling pathway, which further confirmed that abnormal cell proliferation, adhesion, and migration are some of the contributors to the different clinical manifestations of the two subgroups of HNPGL patients. However, we were unable to identify proteins or pathways that were responsible for lymph node metastasis or tumor progression, both of which are key factors in determining clinical treatment and prognosis. In the future, we plan to further improve our classification model to provide support for the development of new treatment plans and prognostic tools for HNPGL patients.

## 5 Conclusion

We conducted a comprehensive analysis of the molecular mechanisms underlying HNPGL and, for the first time, we developed a proteomics-based molecular classification. We applied differential proteomic and functional analyses to various experimental groups to gain insight into the HNPGL proteome and search for meaningful biomarkers as novel therapeutic targets for HNPGL.

## Data availability statement

The data presented in the study are deposited in the ProteomeXchange repository, accession number PXD050299 (https://proteomecentral.proteomexchange.org/cgi/GetDataset?ID=PXD050299).

## Ethics statement

The studies involving humans were approved by each participant provided their informed consent by signing the consent form. The certain consent formula and research protocol were officially reviewed and approved by the Ethics Committee for Human and Animal Research at Peking Union Medical College (no. I-22PJ057). The studies were conducted in accordance with the local legislation and institutional requirements. The participants provided their written informed consent to participate in this study.

## Author contributions

XW: Conceptualization, Formal analysis, Methodology, Writing – original draft. JS: Data curation, Investigation, Methodology, Writing – review & editing. GF: Data curation, Methodology, Writing – review & editing. XT: Formal analysis, Validation, Writing – review & editing. YZ: Methodology, Supervision, Writing – review & editing. ZG: Formal analysis, Methodology, Project administration, Writing – review & editing. WS: Formal analysis, Methodology, Project administration, Writing – review & editing.

## Footnotes

^1^ https://www.pantherdb.org/

^2^ https://cn.string-db.org/
